# Feasibility of hand grip tests during and after hospitalization in geriatric patients: an observational study

**DOI:** 10.1186/s12877-024-05305-6

**Published:** 2024-08-24

**Authors:** Myrthe M Swart, Ligaya Smetsers, Ivan Bautmans, Hugo Plácido da Silva, Merle Geerds, Rudi Tielemans, René Melis, Geeske Peeters

**Affiliations:** 1 Department of Geriatrics, Radboud University Medical Center, Nijmegen, The Netherlands; 2 Gerontology Department, Faculty of Medicine and Pharmacy, Vrije Universiteit Brussel, Brussels, Belgium; 3 Instituto de Telecomunicações, Instituto Superior Técnico, Lisbon, Portugal; 4 ZGT Academy, Ziekenhuisgroep Twente, Almelo, The Netherlands; 5 Biomedical Signals and Systems, Faculty of Electrical Engineering, Mathematics, and Computer Science, University of Twente, Enschede, The Netherlands; 6 UniWeb, Meise, Belgium; 7 Radboudumc Alzheimer Center, Radboud University Medical Center, Nijmegen, The Netherlands

**Keywords:** Grip strength, Grip work, Self-monitoring, Recovery

## Abstract

**Background:**

Monitoring the recovery trajectory during and after hospitalization can be a valuable method to observe whether additional care is needed to optimize recovery. Hand grip strength tests are commonly used to measure an individual’s physical condition. Eforto® is a system to monitor hand grip strength and grip work as measures of recovery. We examined the feasibility of daily repeated hand grip tests measured with Eforto® in geriatric inpatients, during hospitalization and at home after discharge.

**Methods:**

Geriatric inpatients (*n* = 191) were evaluated for grip strength and grip work with Eforto®, twice daily during their admission. We calculated attempt and success rates. Participants were divided into complete, high, moderate, and low attempt/success rate groups to study differences in patient characteristics. Reasons for non-attempt and unsuccessful tests were categorized and analyzed. Nine participants were interviewed about acceptability and user experience within the hospital setting. Four out of twenty participants accepted the invitation to continue the measurements after discharge at home for 4 weeks and were interviewed about acceptability and user experience.

**Results:**

Across the 191 participants, the attempt rate was 85% and 86% of the attempted tests was successful. The main reasons for non-attempt were that the patient felt physically unwell (41%), and that the patient was otherwise engaged, for example receiving care or undergoing medical tests (40%). Measurements were unsuccessful mostly because of the patient not having enough strength to reach the 80% threshold needed for the grip work test (60%). Participants in the complete and high attempt/success rate groups had a shorter length of stay (*p*<0.05) and a lower mortality (*p*<0.05) than participants in the moderate/low groups. The interview data showed good acceptability and user experience during hospitalization. The acceptability was strengthened by experienced usefulness. Self-monitoring at home resulted in low inclusion rate (20%) and low success rate (25%), with the uncertain time after discharge from the hospital as the main barrier.

**Conclusions:**

For most patients, the tests were feasible in the supervised hospital setting. At-home testing with Eforto® is challenging, primarily because of the uncertain time after discharge from the hospital.

## Background

Annually, three to four out of every ten adults of 80 years and older are admitted to the hospital in the Netherlands [1]. The impact of hospitalization on older adults can be high. Frailty is a static concept of a condition characterized by a reduced physiological reserve resulting from the cumulative effects of aging, disabilities and chronic diseases [2]. Due to frailty, there is a risk for prolonged hospitalization, complications during and after hospitalization, and insufficient recovery [3, 4]. Monitoring the recovery trajectory during and after hospitalization can be a valuable method to observe whether additional care is needed to optimize recovery.

Several methods for recovery monitoring have been examined, often using repeated measurements. For example, the number of steps per day is an indicator of recovery in frail older adults, but not in non-frail older adults [5]. Daily questionnaires and an activity tracker, used as recovery indicators, were shown to be feasible in older adults during hospitalization, but this was burdensome for the more frail patients [6]. Additional methods are needed to monitor recovery of older adults containing the key requirements, namely repeated measures during and after hospitalization, feasible in frail and non-frail older adults.

In research and clinical settings, hand grip strength tests are commonly used to measure an individual’s physical condition [7–11]. Although grip strength measures only the strength of the hand and arm muscles, it is associated with global muscle strength [12] and is a key element in the definition of sarcopenia [13]. On a bigger scale, grip strength is a well-known indicator of frailty used in the Fried frailty criteria [14]. Furthermore, grip strength can be used as a predictor for adverse health outcomes following acute health events [15–17]. This suggests that hand grip strength tests are a potential monitoring method for the recovery trajectory.

Three measures of handgrip performance can be differentiated: maximal grip strength (GSmax), fatigue resistance (FR), and grip work (GW) [18]. GSmax indicates the maximal hand grip strength someone achieves during a short bout of squeezing. A lower GSmax is associated with worse daily functioning, more chronic inflammation [8], and a higher rate of mortality [19]. FR indicates how long someone can squeeze maximally until the strength drops below 50% of its maximum. GW combines GSmax and FR as the area under the time-strength curve [18]. A lower GW score is associated with higher fatigue, inflammation, and worse mobility [18, 20, 21]. Compared to GSmax, FR and GW are more responsive to changes in inflammatory status during hospitalization [22, 23]. Therefore, monitoring changes in GW during and after hospitalization may provide useful information on the rate of clinical improvement.

Recently, the innovative e-health system Eforto® was developed and validated to measure GSmax and GW [24]. Eforto® consists of a pneumatic dynamometer that connects automatically to a smartphone app via Bluetooth. The Eforto® system allows for easy data capturing by a healthcare professional (test guided by a professional and the app) or during self-assessment (test guided only by the app), with remote monitoring by a healthcare professional. Reliability and validity have been demonstrated in young and older adults and in hospitalized geriatric patients [24].

An important requirement for grip strength monitoring in clinical settings is that tests should be doable for the target group in clinical practice, either independently or under supervision. The tests should cover the recovery trajectory during and after hospitalization and, thus, ideally, the patients must be able to perform the test themselves after discharge, if needed with the help of informal caregivers. Among geriatric patients, known challenges are hearing loss, cognitive problems such as delirium, and low digital literacy [25–27]. This could complicate the understanding of instructions and the execution of the test.

This study examined the feasibility (specifically usability, user experience, and acceptability) of daily repeated GW tests, measured with Eforto®, for geriatric inpatients. We studied the completion rates, enablers, and barriers of the daily repeated GW tests during and after hospitalization.

## Methods

### Study sample

The current analysis used data from participants in the ongoing Geriatric Resilience Registry (*N* = 285, protocol number 2021-13022) and the FORTO study (*N* = 159, protocol number NL77879.091.21). These studies followed the same protocol for all relevant aspects for the current analysis. The study sample for this research was obtained at the Radboud university medical center, Nijmegen, The Netherlands. From September 2021 to September 2023, all new admissions to the geriatrics ward who met the eligibility criteria were consecutively recruited. The eligibility criteria were: age 65 years or over; baseline measurements could be completed within 48 hours after admission (72 hours when admitted during weekends); speaking and understanding Dutch; no contact isolation; no severe cognitive impairment during the 48 hours (or 72 hours in weekends) of the inclusion time frame (diagnosed by treating physician during comprehensive geriatric assessment); no low-stimulus care; and a life expectancy of more than two weeks. Baseline measurements were part of routine care. All participants with a baseline measurement were approached to take part in our study with repeated daily measurements (RM group), for which separate, written informed consent was asked. Exclusion criteria for participation in daily measurements were being physically unable to squeeze the Eforto® bulb (e.g. paralyzed) and an expected hospital stay of less than three days.

The Geriatric Resilience Registry was reviewed by the research ethics committee of the Radboud university medical center and falls outside the remits of the Medical Research Involving Human Subjects Act. The main aim of FORTO is to develop the Eforto® device and evaluate the predictive value of GW in clinical settings and therefore the Medical Device Regulations apply. FORTO was reviewed by the East Netherlands Research Ethics Committee (METC Oost-Nederland). The ethics committees approved the studies based on the Dutch Code of Conduct for Health Research, the Dutch Code of Conduct for Responsible Use, the Dutch Personal Data Protection Act, and the Medical Treatment Agreement Act. All participants provided oral informed consent, and all participants taking part in repeated daily measurements provided written informed consent as required following Dutch regulations.

### Study design and procedures

We investigated baseline patient characteristics at admission as part of routine care. A combination of validated questionnaires was administered and one Eforto® test was executed. Additional data were extracted from the electronic patient record. Repeated GW measurements were conducted by trained researchers twice daily, i.e., every morning and every afternoon on weekdays. GW measurements were conducted twice daily with the aim to capture variability over time as a potential measure of resilience (recovery capacity) of the system (patient) to adjust to stressors, e.g. acute illness [28, 29]. These analyses are beyond the scope of this paper.

Participants only taking part in the baseline measurement and not in repeated measurements are referred to as the baseline only (BO) group. Participants taking part in the repeated measurements are referred to as the repeated measurements (RM) group.

### Grip measurements

All grip performance measurements (GW, GSmax and FR), were performed using Eforto® with the dominant hand. Following the protocol utilized in previous studies, participants first squeezed three times maximally with 30 seconds rest intervals, and the best values was considered as GSmax (in kPa) [24, 30–32]. Next, after 30 seconds rest, participants squeezed again maximally and maintained this maximal effort for as long as possible. The time (in seconds) until the GS dropped below 50% of its maximum was noted as FR. GW was calculated as GW = 0.75 × GSmax × FR (in kPa*s) as previously described [18]. For the FR and GW test to be considered valid, participants had to reach at least 80% of the GSmax within the first 5 seconds of the test. When this threshold was not reached, the test was stopped and repeated after 30 seconds rest. In case of three insufficient attempts, the test was aborted.

### Usability, user experience, and acceptability of hand grip tests in hospital

We evaluated the usability of the Eforto® tests during admission. We created an overview of all planned, attempted, and successful tests. We omitted the times no researcher was available (such as weekends and holidays) from the denominator. “Planned tests” were defined as those that should have been attempted according to the measurement protocol, i.e., twice daily during admission (Table 1). Due to variations in length of stay and the availability of a researcher, the total number of planned tests differed across participants. “Attempted tests” refer to measurements started according to the measurement protocol. In practice, this meant that the participant had to have (or tried to have) squeezed the Eforto® bulb at least once. A test was rated “successful” if a valid GW result was obtained.

**Table 1:** Definition of planned, attempted and successful tests

Planned tests	All tests that should have been done in accordance with the measurement protocol
Attempted tests	Tests started in accordance with the measurement protocol, either successful or failed. The test should at least provide a GSmax result.
Successful tests	Tests yielding a valid GW result

In the RM group, we investigated the reasons for tests that were not attempted or lack of success in GW tests. We obtained reasons for non-attempt by categorizing open text fields, namely: the patient felt physically unwell; the patient was not instructible; the patient was otherwise engaged (care was given, patient was nursed in isolation due to infection); technological problems; and other/unknown. We categorized reasons for unsuccessful tests as follows: insufficient strength to achieve GW; the patient was not instructible; technological problems; and other/unknown.

From April to July 2022, we recruited participants of the RM group for a short semi-structured interview about Eforto®. The interview took place at least two days after the baseline measurement. In this way, the participant has had a minimum of four measurements with Eforto® giving them the opportunity to state a reliable opinion on the device. Questions were based on the qualitative Unified Theory of Acceptance and Use of Technology (UTAUT) and included advantages and disadvantages of the Eforto® device, experienced barriers, usefulness, and user-friendliness [33].

### Usability, user experience, and acceptability of hand grip tests at home

In a sub study, we piloted the feasibility and acceptability of home testing after discharge. Consecutive recruitment took place among participants in the RM group from mid-April to the beginning of August 2023, until 20 participants were approached. Reasons for non-participation were noted. Home measurements consisted of continued hand grip tests after discharge for four weeks with a planned frequency of two tests per week, using the Eforto® device connected to the participant’s own smartphone. These tests were guided by the Eforto® app with verbal and written instructions. The app has a dedicated setting for self-testing in which the app guides the participant through the steps of the measurement. The app logged the number of tests that were done. Informal caregivers were actively involved when they were available to help and motivate the participant using the self-testing setting of the Eforto® app.

Before discharge, participants (and their informal caregiver, when applicable) received instructions from a trained researcher about self-testing with the Eforto®, by executing one self-test together with the trained researcher. One week after discharge, the participants were called to discuss any problems or questions. After four weeks, a short semi-structured interview took place, based on the UTAUT, about the participants’ experience using the device at home within those of the 20 persons who took the Eforto® device home [33]. Questions included advantages and disadvantages of self-testing at home, experienced barriers, usefulness, and user-friendliness.

### Baseline measures and score calculations

The Clinical Frailty Scale (CFS), developed as a 9-point scale [34], was retrieved from the electronic patient record. The Frailty Index (FI) was based on 35 items from The Older Persons and Informal Caregivers Survey Minimum DataSet (TOPICS-MDS) questionnaire [35]. The scores of (instrumental) activities of daily living ((i)ADL) derived from the TOPICS-MDS (i)ADL questions; higher scores indicate more dependency [36]. The multimorbidity result is the sum of diseases present at admission, used from the TOPICS-MDS, scored on 17 items [36]. The number of medications at home was registered at admission. Health-related quality of life is based on the EuroQoL-5D; higher scores indicate better quality of life [37]. Psychological well-being is a score based on five questions of the mental health domain of the 36-Item Short Form Health Survey (SF-36) questionnaire; higher scores indicate better well-being [38]. Treatment focus is decided on as part of routine care jointly by the patient and geriatrician, and derived from the medical records. At admission, clinicians indicate the treatment focus by assigning the patient to one of the following four categories: 1) curative, meaning fully curative treatment; 2) recovery-oriented care, meaning a treatment focused on recovery knowing the limited expected lifespan; 3) symptom-oriented care, meaning a treatment focused on symptoms knowing the limited expected lifespan; and 4) end of life care, meaning a life expectancy of less than two weeks (excluded in this study). Age, sex, BMI, medical history, medication, treatment focus, and length of stay were extracted from the electronic patient record.

### Statistical analysis

Sample characteristics were described for the BO and RM groups. Attempt and success rates of Eforto® tests were described overall and for each group separately. Reasons for non-attempt and unsuccessful tests were categorized based on open text fields. The RM group participants were divided into four groups based on their attempt rate: 100% attempt (complete); 50%-99% attempt (high); 25%-49% attempt (moderate); and less than 25% attempt (low). We did the same for success rate. We compared patient characteristics between the groups for both attempt and success rates.

Qualitative analyses were conducted for the interview data. We used open coding to identify the enablers and barriers of hand grip tests. This was done independently by two researchers. Codes were compared and revised until consensus was achieved. The codes were further analyzed thematically, and the themes were used to complement and explain the quantitative results.

## Results

**Fig. 1:**
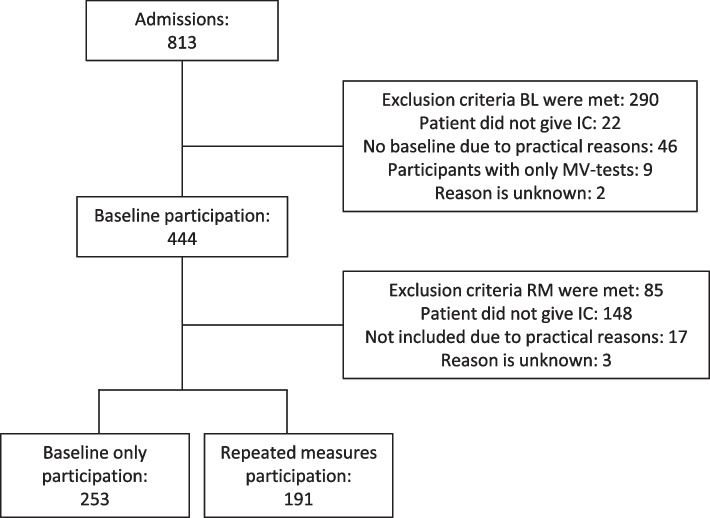
Flow chart of recruitment and inclusion. IC: Informed Consent. BL: baseline. MV: Martin Vigorimeter. RM: Repeated measurements. BL exclusion criteria were life expectancy of <2 weeks, insufficient cognitive function, not speaking Dutch, contact isolation, severe cognitive impairment, low-stimulus care, no baseline measurement possible within 48h of admission (excluding weekends). RM exclusion criteria were expected hospital stay of <3 days, not being able to squeeze

In this study, 813 patients were admitted to the geriatrics ward during the inclusion period (Fig. 1). We included 444 participants; 253 in the BO group and 191 in the RM group, based on their willingness to participate in the repeated measures. 290 patients who did not meet inclusion criteria (i.e. a life expectancy of less than two weeks, insufficient cognition, not speaking Dutch, hospitalized in contact isolation or receiving low-stimulus care) were excluded from baseline measurements. 79 additional patients were excluded due to not consenting, practical reasons, using the Martin Vigorimeter instead of the Eforto® device for GW measurements or unknown reasons. After baseline measurements, 85 patients not meeting inclusion criteria (i.e. an expected hospital stay of less than three days and inability to squeeze) were additionally excluded from the repeated measurements. In addition, 168 patients were excluded for not consenting, practical reasons or unknown reasons. Of the total study sample, 267 participants (60%) were female and the median age was 82.5 years [IQR 77.1, 88.7] (Table 2).

**Table 2:** Patient characteristics

		**Baseline Only (BO)**, *N* = 253		**Repeated Measurements (RM)**, *N* = 191		***p*-value**
**Sex (female)**	n (%)	171 (68%)		96 (50%)		<0.001
**Age (years)**	Median [IQR]	83.0 [77.7, 88.8]		82.0 [76.6, 87.9]		0.31
**CFS**						0.26
Fit (CFS 1-3)	n (%)	29 (12%)		20 (10%)		
Mild to moderate (CFS 4-6)	n (%)	95 (38%)		87 (46%)		
Severe (CFS 7-9)	n (%)	127 (51%)		84 (44%)		
**Frailty Index**	Median [IQR]	0.3 [0.3, 0.4]		0.3 [0.3, 0.4]		0.68
**Multimorbidity**	Median [IQR]	4.0 [3.0, 6.0]		4.0 [3.0, 6.0]		0.57
**Nr. of medications prior to admission**	Median [IQR]	11.0 [7.0, 14.0]		10.0 [7.0, 14.0]		0.62
**EuroQol 5D**	Median [IQR]	0.4 [0.2, 0.7]		0.4 [0.2, 0.6]		0.38
**Psychological well-being**	Median [IQR]	76.0 [60.0, 88.0]		76.0 [60.0, 88.0]		0.57
**(i)ADL before admission**	Median [IQR]	24.0 [17.5, 30.5]		24.0 [18.0, 30.0]		0.98
**(i)ADL at admission**	Median [IQR]	36.0 [28.0, 38.0]		37.0 [30.0, 39.0]		0.36
**Treatment focus**						0.14
Curative	n (%)	40 (16%)		36 (19%)		
Recovery-oriented care	n (%)	183 (75%)		145 (77%)		
Symptom-oriented care	n (%)	22 (9.0%)		8 (4.2%)		
**Length of stay (days)**	Median [IQR]	7.0 [4.0, 11.0]		9.0 [6.0, 13.0]		<0.001
**Deceased during admission**	n (%)	10 (4.0%)		5 (2.6%)		0.60
		**Men,** *N* = 82	**Women,** *N* = 171	**Men,** *N* = 95	**Women,** *N* = 96	
**GSmax at baseline (kPa)**	Median [IQR]	46 [38, 55]	33 [26, 44]	48 [37, 59]	34 [26, 45]	M: 0.47 W: 0.70
**FR at baseline (s)**	Median [IQR]	26 [17, 41]	25 [15, 36]	24 [17, 32]	19 [13, 32]	M: 0.20 W: 0.03
GW at baseline (kPa*s)	Median [IQR]	1070 [670, 1485]	621 [397, 1001]	936 [619, 1166]	458 [306, 1055]	M: 0.08 W: 0.30

BO group: Participants taking part only in baseline measurement. RM group: participants taking part in repeated measurements. CFS (Clinical Frailty Scale): 1-9, lower is better. Frailty index: 0-1, lower is better. Multimorbidity: 0-17, lower is better. EuroQol 5D: -0.4-1, higher is better. (i)ADL: (instrumental) Activities of Daily Living, 5-40, higher scores indicate more dependency. Psychological wellbeing: 0-100, higher is better. GSmax: maximal Grip Strength, higher is better. FR: Fatigue resistance, higher is better. GW: Grip Work, higher is better

### Usability, user experience, and acceptability of hand grip tests in hospital

Across the 444 participants (Table 2) who completed baseline measurements (BO group and RM group), we have registered 2108 hand grip tests executed with Eforto®. Attempted tests result in a GSmax score and successful tests in a GW score. Data on attempt and success rates were available for 1870 (89% of 2108) planned tests of 391 participants. Of these 1870 planned tests, 222 were baseline measurements in 222 participants (BO group) and 1648 were planned baseline and repeated measurements in 169 participants (RM group). In the BO group, 210 (95% of 222) participants attempted the measurement and 164 (78% of 210 attempted tests) provided a successful GW result. In the RM group, 1409 (85% of 1648) tests were attempted and 1217 (86% of 1409 attempted tests) tests were successful. Non-attempt and unsuccessful tests were equally reported in the morning (non-attempt 42%, unsuccessful 56%) and afternoon (non-attempt 58%, unsuccessful 44%).

The reasons why measurements were not attempted (15% of the planned measurements in the RM group) or not successful (26% of the attempted measurements in the RM group) are listed in Tables 3 and 4. Within the RM group, the main reasons for non-attempt were that the patient felt physically unwell (41%), and that the patient was otherwise engaged, for example receiving care or undergoing medical tests (40%). In 18%, the reason for non-attempt was categorized as other/unknown.

**Table 3:** Reasons for non-attempt

Reasons	Nr. of measurements	% of measurements	Nr. of participants
**Patient felt physically unwell**	99	41	48
**Patient was otherwise engaged**	96	40	52
**Technological problems**	2	1	2
**Other/unknown**	42	18	25

Percentages are given relative to the 239 planned but not attempted measurements (GS, FR and GW) in the group scheduled to be measured twice daily (RM group). Participants had multiple measurements planned and can therefore end up in multiple rows. Total number of participants with non-attempted tests was 87

**Table 4:** Reasons for unsuccessful results

Reasons	Nr. of measurements	% of measurements	Nr. of participants
**Not enough strength to achieve 80% threshold for GW**	116	60	46
**Technological problems**	5	3	5
**Patient was not instructible**	3	2	2
**Other/unknown**	68	35	34

Percentages are given relative to the 192 attempted but unsuccessful measurements (GW) in the group scheduled to be measured twice daily (RM group). Participants have attempted multiple measurements and can therefore end up in multiple rows. Total number of participants with unsuccessful tests was 75

Measurements were unsuccessful mostly because of the patient not having enough strength to reach the 80% threshold needed for the GW test (60%). Other reasons were technological problems (3%) and the patient not being instructible to squeeze as long as possible (2%). In 35% of the unsuccessful measurements, the reason for not achieving a GW result was unknown or categorized as other.

Nine participants were interviewed about the Eforto® measurements during hospitalization and data saturation was achieved. All participants found Eforto® easy to use and found the provided guidance sufficient and clear (usability): *“It is just grabbing and squeezing a small ball, nothing more than that”* (male, 73). Doing two measurements a day was acceptable, and the supervised measurements were doable (acceptability, user experience). For the majority of the participants, the aim of Eforto® was not clear. The participants thought that Eforto® could be implemented in the hospital under the condition that Eforto® does have value and that it is useful to add to healthcare. A potential obstacle of the Eforto® measurements during hospitalization could be available time. Measurements should fit in the patients’ daily planning as the measurements do take some time. One participant did not want to do measurements when he felt too tired and one participant mentioned that Eforto® is not suitable for patients who are too old and in the terminal phase (acceptability): *“Then you are at your end. Then I do not need it anymore.”* (female, 91). Overall, opinions and experiences with Eforto® differ per patient.

### Comparison of patient characteristics based on attempt and success rate

**Fig. 2:**
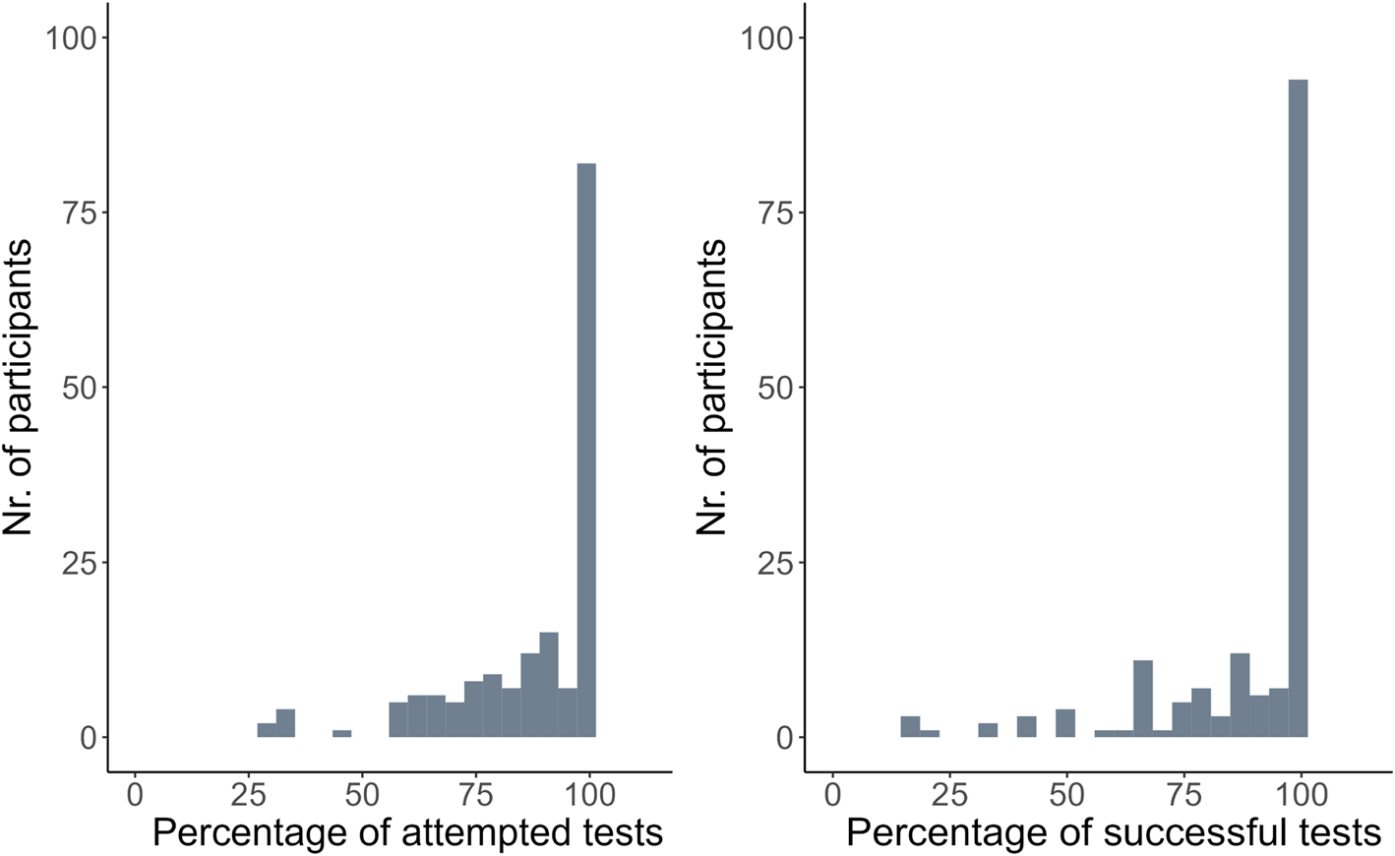
Distribution of attempt and success rates within the RM group. Left: distribution of attempt rate. Right: distribution of success rate

Half of participants (49%) attempted all tests and 96% attempted at least half of the scheduled tests. (Figure 2 and Table 5). Attempt rates below 25% were not observed. The other three groups differed significantly in scores for psychological well-being (*p* < 0.01, Table 5). Participants with moderate attempt rates had the worst scores for psychological well-being. Treatment focus was significantly different between the groups (*p* < 0.05). The group with moderate attempt rates had the highest percentage of participants with a curative treatment. Statistically significant differences in length of stay were found (*p* < 0.001). The complete group had the shortest length of stay of 7 [IQR 4, 11] days. Mortality was higher in the moderate (17%) and the high attempt group (5%) compared to the complete attempt group (0%, *p* < 0.05). Participants having a moderate attempt rate more often had a patient-specific reason (i.e. feeling physically unwell; 80%) compared to participants in the high group (34%) (analysis not shown in table). However, only a small number of participants had a moderate attempt rate (*n*=7 (4%)).

**Table 5:** Comparison of patient characteristics based on attempt rates within the RM group

		**Complete**, *N* = 82		**High**, *N* = 80		**Moderate**, *N* = 7		***p*-value**
**Sex (female)**	n (%)	43 (52%)		38 (48%)		5 (71%)		0.44
**Age (years)**	Median [IQR]	82.5 [78.2, 87.9]		81.4 [76.4, 87.2]		82.0 [78.3, 89.6]		0.89
**CFS**								0.46
Fit (CFS 1-3)	n (%)	7 (8.5%)		10 (13%)		2 (29%)		
Mild to moderate (CFS 4-6)	n (%)	36 (44%)		38 (48%)		2 (29%)		
Severe (CFS 7-9)	n (%)	39 (48%)		32 (40%)		3 (43%)		
**Frailty Index**	Median [IQR]	0.4 [0.3, 0.4]		0.3 [0.3, 0.4]		0.3 [0.3, 0.5]		0.72
**Multimorbidity**	Median [IQR]	4.0 [3.0, 5.0]		4.0 [3.0, 6.0]		4.0 [3.0, 5.5]		0.40
**Nr. of medications prior to admission**	Median [IQR]	11.0 [7.0, 14.0]		10.0 [6.8, 14.0]		4.0 [3.5, 15.0]		0.61
**EuroQol 5D**	Median [IQR]	0.5 [0.2, 0.6]		0.4 [0.2, 0.6]		0.4 [0.2, 0.6]		0.69
**Psychological well-being**	Median [IQR]	80.0 [61.0, 92.0]		76.0 [52.0, 88.0]		48.0 [28.0, 56.0]		0.006
**(i)ADL before admission**	Median [IQR]	25.0 [17.3, 32.0]		23.5 [18.0, 28.3]		24.0 [19.5, 35.0]		0.50
**(i)ADL at admission**	Median [IQR]	36.5 [31.0, 39.0]		37.0 [33.0, 40.0]		37.0 [34.0, 38.5]		0.44
**Treatment focus**								0.04
Curative	n (%)	13 (16%)		15 (19%)		4 (57%)		
Recovery-oriented care	n (%)	62 (77%)		63 (80%)		3 (43%)		
Symptom-oriented care	n (%)	6 (7.4%)		1 (1.3%)		0 (0%)		
**Length of stay (days)**	Median [IQR]	7.0 [4.0, 11.0]		10.5 [8.0, 16.0]		8.5 [8.0, 9.0]		< 0.001
**Deceased during admission**	n (%)	0 (0%)		4 (5.0%)		1 (17%)		0.02
		**Men,** *N* = 39	**Women,** *N* = 43	**Men,** *N* = 42	**Women,** *N* = 38	**Men,** *N* = 2	**Women,** *N* = 5	
**GSmax at baseline (kPa)**	Median [IQR]	50 [36, 57]	34 [25, 46]	49 [39, 64]	31 [26, 44]	42 [33, 52]	28 [27, 38]	M: 0.52 W: 0.23
**FR at baseline (s)**	Median [IQR]	24 [18, 30]	19 [13, 29]	24 [15, 31]	22 [15, 37]	15 15 [15, 15]	28 [20, 41]	M: 0.11 W: 0.46
GW at baseline (kPa*s)	Median [IQR]	783 [614, 1153]	432 [293, 889]	952 [611, 1161]	625 [323, 1055]	686 [686, 686]	567 [420, 1169]	M: 0.45 W: 0.42

Complete group: patients having 100% attempted tests. High group: patients having 50%-99% attempted tests. Moderate group: patients having 25%-49% attempted tests. Low group: patients having 25% or less attempted tests (no participants in this group)

CFS (Clinical Frailty Scale): 1-9, lower is better. Frailty index: 0-1, lower is better. Multimorbidity: 0-17, lower is better. EuroQol 5D: -0.4-1, higher is better. (i)ADL: (instrumental) Activities of Daily Living, 5-40, higher scores indicate more dependency. Psychological wellbeing: 0-100, higher is better. GSmax: maximal Grip Strength, higher is better. FR: Fatigue resistance, higher is better. GW: Grip Work, higher is better

Over half of participants (56%) successfully completed all scheduled tests, with the majority of participants (88%) successfully completing at least half of the scheduled tests (Fig. 2 and Table 6). The number of medications prior to admission was significantly different between the groups (*p* < 0.05), with the complete group having the lowest number of medications (median 10, [IQR 7, 13] medications). Length of stay was significantly different between the groups (*p* < 0.05), with the complete group having the shortest length of stay (median 8, [IQR 5, 11] days). The moderate success rate group had the highest mortality of 17% (*p* < 0.05). Participants having a low success rate more often had a patient-specific reason (i.e. not having enough strength; 75%) compared to participants in the moderate and high group (54%) (analysis not shown in table). However, only a small number of participants had a low success rate (*n*=12 (7%)).

**Table 6:** Comparison of patient characteristics based on success rates within the RM group

		**Complete**, *N* = 94		**High**, *N* = 54		**Moderate**, *N* = 9		**Low**, *N* = 12		***p*-value**
**Sex (female)**	n (%)	45 (48%)		30 (56%)		4 (44%)		7 (58%)		0.78
**Age (years)**	Median [IQR]	82.7 [75.7, 88.6]		81.7 [78.9, 87.0]		81.1 [76.3, 90.0]		79.0 [72.9, 85.2]		0.65
**CFS**										0.36
Fit (CFS 1-3)	n (%)	14 (15%)		5 (9.3%)		0 (0%)		0 (0%)		
Mild to moderate (CFS 4-6)	n (%)	38 (40%)		29 (54%)		5 (56%)		4 (33%)		
Severe (CFS 7-9)	n (%)	42 (45%)		20 (37%)		4 (44%)		8 (67%)		
**Frailty Index**	Median [IQR]	0.3 [0.3, 0.4]		0.4 [0.2, 0.4]		0.4 [0.3, 0.5]		0.3 [0.3, 0.4]		0.53
**Multimorbidity**	Median [IQR]	4.0 [3.0, 6.0]		4.0 [3.0, 6.0]		5.0 [4.0, 7.0]		4.0 [3.0, 5.0]		0.25
**Nr. of medications prior to admission**	Median [IQR]	10.0 [7.0, 13.0]		11.5 [6.0, 14.0]		15.0 [14.0, 17.0]		12.5 [9.5, 16.0]		0.02
**EuroQol 5D**	Median [IQR]	0.4 [0.2, 0.6]		0.4 [0.1, 0.6]		0.1 [0.1, 0.4]		0.6 [0.3, 0.7]		0.23
**Psychological well-being**	Median [IQR]	76.0 [60.0, 88.0]		76.0 [56.0, 84.0]		68.0 [52.0, 84.0]		80.0 [57.0, 96.0]		0.54
**(i)ADL before admission**	Median [IQR]	24.0 [18.0, 31.0]		24.0 [18.0, 30.0]		27.0 [21.0, 35.0]		24.0 [23.0, 26.0]		0.78
**(i)ADL at admission**	Median [IQR]	37.0 [32.0, 39.0]		37.0 [33.3, 39.0]		37.0 [32.0, 38.0]		32.5 [27.8, 37.8]		0.51
**Treatment focus**										0.80
Curative	n (%)	21 (22%)		7 (13%)		1 (11%)		3 (25%)		
Recovery-oriented care	n (%)	68 (72%)		43 (83%)		8 (89%)		9 (75%)		
Symptom-oriented care	n (%)	5 (5.3%)		2 (3.8%)		0 (0%)		0 (0%)		
**Length of stay (days)**	Median [IQR]	8.0 [5.0, 11.0]		10.5 [7.3, 15.8]		12.0 [4.0, 14.0]		9.0 [6.5, 11.3]		0.04
**Deceased during admission**	n (%)	2 (2.2%)		0 (0%)		1 (11%)		2 (17%)		0.01
		**Men,** *N* = 49	**Women,** *N* = 45	**Men**, *N* = 24	**Women**, *N* = 30	**Men**, *N* = 5	**Women**, *N* = 4	**Men**, *N* = 5	**Women**, *N* = 7	
**GSmax at baseline (kPa)**	Median [IQR]	50 [39, 61]	36 [26, 48]	51 [39, 59]	34 [26, 44]	50 [45, 54]	33 [30, 38]	35 [23, 38]	27 [20, 29]	M: 0.16 W: 0.30
**FR at baseline (s)**	Median [IQR]	24 [18, 32]	22 [15, 37]	24 [18, 27]	18 [12, 26]	13 [12, 14]	11 [11, 11]	NA	NA	M: 0.12 W: 0.08
GW at baseline (kPa*s)	Median [IQR]	855 [614, 1172]	567 [330, 1169]	963 [618, 1072]	419 [310, 753]	731 [647, 815]	289 [289, 289]	NA	NA	M: 0.72 W: 0.27

Complete group: patients having 100% successful tests. High group: patients having 50%-99 successful tests. Moderate group: patients having 25%-49% successful tests. Low group: patients having 25% or less successful tests (no participants in this group)

CFS (Clinical Frailty Scale): 1-9, lower is better. Frailty index: 0-1, lower is better. Multimorbidity: 0-17, lower is better. EuroQol 5D: -0.4-1, higher is better. (i)ADL: (instrumental) Activities of Daily Living, 5-40, higher scores indicate more dependency. Psychological wellbeing: 0-100, higher is better. GSmax: maximal Grip Strength, higher is better. FR: Fatigue resistance, higher is better. GW: Grip Work, higher is better

### Usability, user experience, and acceptability of hand grip tests at home

Twenty participants (13% out of 159 included participants) were approached for self-testing after discharge, of whom four participants actually agreed to take part. The four participating individuals had a mean age of 79 years (min. 72, max. 92), 50% were female and the mean CFS was 3.3 (min. 1, max. 6). They were instructed to do the hand grip tests twice a week at home for four weeks. One participant, female and 77 years old, succeeded in completing seven successful tests. The three other participants had several reasons for not completing the tests: one participant attempted the GS measurements but stopped before the GW test. One participant tried her best even with help of a family member, but could not manage to do the test. One participant did no tests at all because he did not find it useful. The offered support by phone was not used by the participants.

The sixteen individuals who chose not to participate had a mean age of 84 years (min. 76, max. 93), 60% was female and the mean CFS was 4.5 (min. 2, max. 7). Six could not participate due to practical reasons (i.e. discharged too quickly to inform and recruit, or lived too far from the hospital; ownership of smartphone unknown). Four did not have access to a smartphone. Five did have a smartphone, but chose not to participate as they felt that it would take too much time and effort in the uncertain circumstances after discharge. One had a smartphone, but thought they would not be able to do the home tests because of the digital literacy needed. Help of an informal caregiver was discussed with each individual, but this did not change the choice for participation.

Enablers mentioned in the interviews were support of a family member and having insight into your own progress after discharge. The Eforto® system was found interesting and the measurements were generally easy and quick. The guidance of the smartphone application ensured an easy measurement flow: “*It is automated”* (female, 77). The main barrier for patients to participate in the home measurements was the uncertain time after discharge from the hospital. Some patients had to rehabilitate before going home, or had to start up home care. Another barrier was digital literacy. Older adults often have no smartphone and when they do, they lack the skills to quickly learn using a new app, e.g.: *“I must have pressed the wrong button. That doesn’t surprise me.”* (female, 77).

The participants had different ideas about the user experience and user-friendliness of the Eforto® device. While one was mostly positive and would advise this device to others, another thought the use of the app was too difficult and would therefore not recommend using it. Similar results were found for motivation, adherence, and willingness to continue using the Eforto® device. Some participants had difficulties doing the tests twice a week: *“Twice per week really is more than enough”* (male, 72); *“You don’t always feel like doing the test”* (male, 72). Participants differed in opinions about the usefulness of the device. One person experienced no usefulness at all, while others mentioned muscle training and insight in recovery as positive aspects of Eforto®.

## Discussion

This study examined the usability, user experience, and acceptability of daily repeated hand grip tests, measured with Eforto®, in hospitalized geriatric patients and after discharge. For in-hospital usability, we found that guided repeated hand grip tests were attempted in 85% (RM group) to 95% (BO group) of the planned test moments during hospitalization. Attempted tests always result in a GSmax score. A GW result was achieved in 78% (BO group) to 86% (RM group) of the attempted tests. The feasibility of GW tests for geriatric inpatients has among others been described in a study of 46 hospitalized geriatric patients aged 70 years and older. These participants were all able to execute the GS and GW tests, giving attempt and success rates of 100% [39]. The BO group in our study, with comparable sample characteristics and test protocol, had a similar attempt rate (95%), but lower success rate (78%) compared to the previous study. The lower success rate may reflect that our study sample is sicker or more frail than the study sample described in the previous study.

Recovery monitoring can be studied with different methods, for example using wearables [40], bedside observations [41], and questionnaires [42], all having different challenges for feasibility. Wearables have the advantage of continuous monitoring without any effort for the patient. However, the interpretation of the results may be complex, while the GW tests provide a direct, easy-to-interpret result. In a study by Hubbard *et al.*, professionals monitored routine mobility and balance as a measure for recovery [41]. While direct monitoring by a professional might reduce the amount of missing data, it is less feasible once a patient is discharged. The hand grip tests with Eforto® are available for self-testing after discharge to monitor patients’ recovery beyond the period of hospitalization. Questionnaires are a cheap and easy method to monitor recovery over time, but might give more subjective results compared to a standardized squeezing measurement like a GW test with Eforto®. Questionnaires could therefore be valuable as complementary data to the Eforto® results.

Our study sample mainly consists of frail older adults, as shown by the high FI and CFS (Table 2)*.* Only a small group had over 50% non-attempted measurements (7 participants) or over 75% unsuccessful measurements (12 participants). This means that most frail older adults were able to successfully perform the GW test. The main reasons for repeated non-attempt and unsuccessful tests were patient-specific, such as feeling physically unwell or not having enough strength. The 7 and 12 participants with multiple non-attempted measurements or unsuccessful measurements had a longer length of stay and a higher percentage of mortality compared to participants with more attempted/successful tests. Although these results are based on small groups of participants, multiple unsuccessful or not attempted tests point towards poor health or frailty.

Non-attempt of Eforto® tests was often due to patient-specific reasons (41%), but equally often due to availability constraints (40%, patient was otherwise engaged). This category mainly encompasses moments when other caregivers were working at the patient’s bedside. Contrary to the patient-specific reasons, this does not inform us about the patient’s condition, but more about the planning of daily care. Embedding use of the Eforto® device in routine daily care will improve attempt rates.

The interview results regarding user experience and acceptability show that the tests during hospitalization were feasible for most participants. In-hospital feasibility did not depend on the digital literacy of the participants or the possession of a smartphone, because the measurements were done with help of trained staff and devices available within the hospital. Participants emphasized that the goal of Eforto® measurements should be clear to stay motivated. In the future, we aim to feedback the grip test results to the patients and caregivers to inform them about the recovery trajectory of the patient. This will likely boost the perceived usefulness of the measurement and subsequently the experienced usability.

At-home testing with Eforto® resulted in a low inclusion rate (20%) and a low success rate (25%), primarily because of the uncertain time regarding discharge from the hospital. Additionally, we observed a low digital literacy among the geriatric patients. The post-discharge measurements were only suitable for participants having a smartphone. Four of the twenty participants approached for the home measurements could not participate due to not having a smartphone. Over the coming decade, it is expected that the percentage of older adults owning a smartphone will continue to increase, and that their digital literacy will improve, making this less of a barrier. In the meantime, strategies to reach the small group of older adults without a smartphone include asking support from informal caregivers or home care professional or hospitals may be able to lend a smartphone together with the Eforto® device and provide assistance by phone during the measurements. Nicosia *et al.* studied smartphone-based monitoring of cognitive function in older adults and found an adherence rate of 80%, which is much higher than our success rate [43]. In that study, participants without a smartphone were provided one and participants received extensive support during the study in multiple ways. We did offer support by phone during the home testing, but the participants did not make use of it. More support might be needed for home testing with Eforto®.

More importantly, the current context may have negatively influenced the low acceptability of home testing, as there was no direct benefit for the participants in this research setting. Similar to the hospital setting, in the future we aim to feedback the results to the patient and caregiver. The recommendation of a health care professional to use Eforto® will likely improve the motivation and adherence of patients. Although a minority at this point, some participants already acknowledged the value of Eforto® tests at home. These participants had a positive user experience and would like to see it implemented in daily care.

Participants and physicians have made suggestions for further improvement of the Eforto® device. They proposed to add normative values for correct interpretation of the results, which is an important step towards implementation in daily care. In addition, they asked for more explanation of the results in relation to clinical outcomes. Future research should therefore focus on normative values, on associations of GSmax, FR and GW with clinically relevant outcomes, such as recovery, and on the best way to present test results to patients and (informal) caregivers to inform them about the recovery trajectory.

### Strengths and limitations

This study involves a unique population of older adults with moderate to severe frailty who are prospectively followed during their hospital admission. We established a good sample size and a good representation of the geriatric hospitalized patients for the quantitative analyses. Our analyses were mostly done within the RM group, whom were willing to participate in daily measurements. Participants who were unable to complete the test at baseline were less likely to participate in the repeated measurements. We showed that the RM group is similar to the BO group except for sex and length of stay, but note that an expected length of stay of less than two days was an exclusion criterion for the RM group. These findings suggest that the RM group is representative of patients admitted to the geriatrics ward, except the most frail older adults with a life expectancy of less than two weeks, for whom benefit of recovery monitoring is futile anyway, or severe cognitive impairment who cannot be instructed for the test, which was only a small group in our study. Therefore, we conclude that our findings are generalizable to the group of acutely admitted geriatric patients.

A limitation of this study was the total burden of study participation. For future analysis in the field of complexity science, we plan to study Dynamical Indicators Of Resilience [28, 29], for which we need frequently repeated measurements of GW. We therefore decided to incorporate two daily GW measurements. However, two daily GW measurements combined with some short questionnaires (completed for other research purposes) might have been tiring for frail patients in poor health. This might have led to a lower feasibility of the Eforto® device in this study protocol and setting. In 35% of the unsuccessful tests the reasons were not noted, which could have led to underestimation of patient-specific reasons for unsuccessful tests. Our analyses did not include physical activities that the patient may have done shortly before performing the Eforto® test, such as ADL and physical therapy. These activities, needing energy and strength, may have lowered the feasibility of Eforto® tests.

## Conclusions

In this study we describe the usability, user experience, and acceptability of hand grip strength tests in geriatric patients during and after hospitalization. For most patients, the tests were feasible in the supervised hospital setting: across the 191 participants, the attempt rate was 85% and 86% of attempted tests was successful. At-home testing with Eforto® is challenging, primarily because of the uncertain time after discharge from the hospital and limited digital literacy.

## Data Availability

The data that support the findings of this study are not openly available due to reasons of sensitivity and are available from the corresponding author upon reasonable request.

## Abbreviations

BL: Baseline
BO group: Baseline only group
FI: Frailty index
FR: Fatigue resistance
GSmax: Maximal grip strength
GW: Grip work
(i)ADL: (instrumental) activities of daily living
IC: Informed consent
MV: Martin Vigorimeter
RM group: Repeated measurements group
SF-36: 36-Item Short Form Health Survey
TOPICS-MDS: The Older Persons and Informal Caregivers Survey Minimum DataSet
UTAUT: Unified theory of acceptance and use of technology
